# Expression of Salivary Ceramide Synthase 1 (CERS1) in Recurrent Aphthous Stomatitis (RAS): A Cross-Sectional Institutional Study

**DOI:** 10.7759/cureus.41597

**Published:** 2023-07-09

**Authors:** Sangamithra Surendran, Pratibha Ramani, Karthikeyan Ramalingam, Selvaraj Jayaraman

**Affiliations:** 1 Oral Pathology and Microbiology, Saveetha Dental College and Hospitals, Saveetha Institute of Medical and Technical Sciences, Chennai, IND; 2 Biochemistry and Molecular Biology, Saveetha Dental College and Hospitals, Saveetha Institute of Medical and Technical Sciences, Chennai, IND

**Keywords:** salivary analysis, saliva, apoptosis, ceramide synthase 1, ceramide, aphthous ulcer, recurrent aphthous stomatitis

## Abstract

Background

The increased rate of apoptosis is one of the major causes of ulcer formation. A variety of factors can influence the rate of apoptosis. Ceramide (CER) is one such factor that has been proposed to play a role in signaling apoptosis induced by extracellular agents. Recurrent aphthous stomatitis (RAS) is a common condition that initially presents in children or adolescents. Multiple recurrent small, round, or ovoid ulcers with erythematous haloes and circumscribed margins are its characteristic features. Its pathogenesis is still a mystery. Ceramide synthase 1 (CERS1) aids in the production of C18 CER. Although the role of CERS1 in aphthous is well understood, its possible intricate role in pathogenesis remains unknown.

Aim

To evaluate the expression of salivary CERS1 in patients with RAS and healthy individuals.

Materials and methods

30 patients were included in the present study. Ethical clearance for this study was obtained, and there were no gender or age limits for enrollment. After obtaining informed consent, 30 salivary samples were collected from patients with RAS (n=15) and from healthy individuals (n=15). Enzyme-linked immunosorbent assay (ELISA) was performed using the CERS1 kit by MyBioSource Inc (San Diego, USA) and the results were recorded. The Chi-square test and Independent t-test were used for statistical analysis with SPSS v23.0 (IBM, Chicago, USA) with a significant p-value of <0.05.

Results

CERS1 expression was identified in the saliva of all participants. There was a decrease in the salivary CERS1 level in RAS patients (7.6 +/- 2.0 pg/ml) when compared to healthy individuals (8.3 +/- 1.8 pg/ml) but it did not achieve statistical significance.

Conclusion

We found that salivary CERS1 levels decreased in RAS patients. More research is required to understand CERS1's pathogenetic role.

## Introduction

Recurrent aphthous stomatitis (RAS) is a common condition that is characterized by one or more recurrent small, round to-ovoid ulcers with an erythematous halo and circumscribed margins. They typically present for the first time during childhood [1]. The diagnosis and treatment of these recurrent oral lesions are frequent issues in both general and specialty dental practice, as RAS is the most prevalent ulcerative disease of the oral mucosa [2]. Epidemiologic studies have demonstrated that the population studied, the criteria for diagnosis, and environmental factors affect the prevalence of RAS. The prevalence of RAS in children can reach up to 39% and is influenced by whether one or both parents have the condition. RAS are 90% more likely to develop in children of parents who have them than in children of parents who don't [3]. RAS has an unknown etiology, but a number of local, systemic, immunologic, genetic, allergic, dietary, and microbial factors have been suggested as potential contributors [4-7].

RAS is characterized by recurrent episodes of one or more sore, painful ulcers that are round, and shallow, and occur at intervals of a few days to a few months. All RAS types are uncomfortable recurrent ulcers. On rare occasions, patients experience tingling or burning prodromal symptoms prior to the development of the lesions [8,9]. RAS's pathogenesis could be attributed to cell-mediated inflammation, most likely, T-cells and the production of tumor necrosis factor (TNF) are involved [10]. A comprehensive overview of the pathogenesis of RAS is yet to be developed.

Apoptosis-associated diseases could be associated with prolonged cell survival as noted in cancers, autoimmune diseases, and viral infections. Increased apoptosis is noted in Acquired Immunodeficiency Syndrome (AIDS), neurodegenerative diseases, myelodysplastic syndromes, ischemic injuries and toxins. One of the major causes of any ulcer formation is the increased rate of apoptosis [11,12]. The rate of apoptosis can be affected by factors like ceramide (CER) [13]. CERs are sphingolipids containing fatty acids and sphingosine. CER controls a variety of essential cellular reactions by serving as a crucial mediator in metabolism and signaling pathways [14]. CER has been proposed to play a role in apoptosis induced by extracellular agents such as TNF or the anti-Fas antibody, and CER analogs [13]. TNF has been shown to have a direct cytotoxic effect on gastric epithelial cells in addition to its acute proinflammatory potency. The relationship between CER formation, the induction of apoptosis, and gastric mucosal damage has been studied during the process of gastric ulcer formation [15]. CER is produced by six different ceramide synthases (CERSs). Most of these enzymes are located in the endoplasmic reticulum. Each CERS chooses a different fatty acid chain length for the synthesis of endogenous CER. CERS1 helps to produce the C18 type of CER. Recent studies have found that excessive CERS1 expression impairs cell growth [16]. 

The role of CERS1 in the pathogenesis of RAS is still unclear. The presence of this enzyme in saliva and its role in RAS has not been explored. Hence, we have analyzed the expression of CERS1 in the saliva of both patients with RAS and healthy individuals.

## Materials and methods

This is a cross-sectional institutional study. The participation was entirely voluntary and informed consent was obtained. The sample population was drawn at random from the outpatient department of Saveetha Dental College and Hospitals. The sample size was calculated using G*power 3.1 software. 

Ethical clearance for this study was obtained and there were no gender or age limits for enrollment. The inclusion criteria consisted of patients with an incidence of RAS in the past three months and apparently healthy individuals. Exclusion criteria consisted of patients with RAS who did not have any recent episodes (more than three months). Unstimulated saliva was collected from 30 patients; 15 from patients with RAS and 15 from healthy individuals.

Unstimulated saliva was collected using a collection tube. The foam was excluded and the remaining salivary sample was immediately frozen at -20°C. At the time of evaluation, the collected samples were defrosted and spun at 500 RPM for 10 minutes at 4°C.

**Figure 1:**
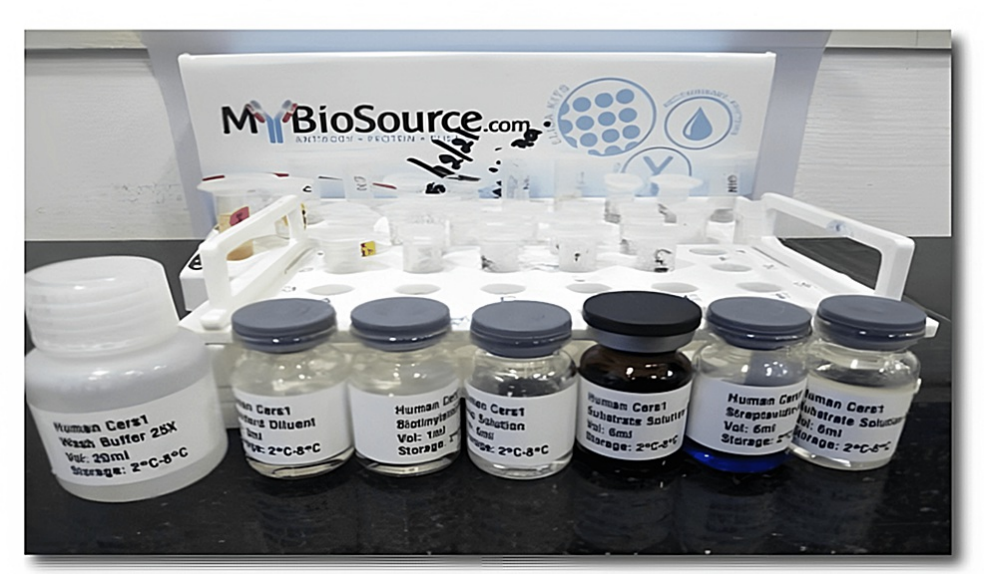
ELISA kit Picture showing the ELISA kit of CERS1 by MyBioSource Inc. ELISA: Enzyme-linked immunosorbent assay; CERS: Ceramide synthase 1

CERS1 levels in saliva were estimated using a CERS1 enzyme-linked immunosorbent assay (ELISA) kit from MyBioSource Inc (San Diego, USA) (Figure 1).

200μl of biotin-antibody was added and the mixture was then incubated at 37°C for one hour. After that, 90μl of 3,3', 5,5;-tetramethylbenzidine (TMB) chromogen solution substrate was added, and it was incubated for 10 to 30 minutes. Finally, using a 450nm microplate reader, the optical density of each well was ascertained in 30 minutes. 

The data were recorded and tabulated. The chi-square test and Independent t-test were used for statistical analysis with SPSS v23.0 (IBM, USA) with a significant p-value of <0.05.

## Results

This cross-sectional institutional study was performed to evaluate the expression of CERS1 in salivary samples of patients with RAS and healthy individuals.

Demographic details of the included patients are as follows: The age range of patients was 22 to 32 years at the time of sample collection, with a mean age of 27 years. There were 17 females and 13 males in our study (Table 1). We found that all of the included subjects had CERS1 in their saliva irrespective of their gender. 

**Table 1:** Gender distribution of study samples Gender distribution revealed that among cases, six males and nine females; and for controls, seven males and eight females were included.

S.No	Gender	Cases	Controls	
1	Male	6 (40%)	7 (46.7%)	p-value = 0.71, Chi-square test
2	Female	9 (60%)	8 (53.3%)	
	TOTAL	15 (100%)	15 (100%)	

Independent t-test showed that the salivary CERS1 level in RAS patients was (7.6 +/- 2.0 pg/ml) when compared to healthy individuals (8.3 +/- 1.8 pg/ml). The result was statistically insignificant (p>0.05) (Table 2).

**Table 2:** Mean values of salivary CERS1 levels This table shows the mean values of salivary CERS1 levels among RAS patients and controls. CERS1: Ceramide synthase 1; RAS: Recurrent aphthous stomatitis

S.No	Measures	Cases	Controls	
1	Mean	7.6	8.3	p-value = 0.69, Independent t-test
2	SD	2	1.8	

**Figure 2:**
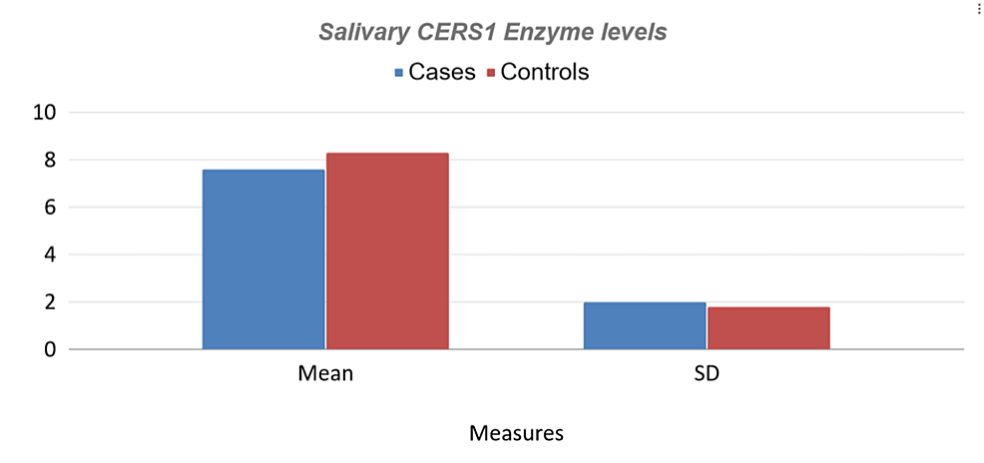
Graphical representation of enzyme levels The graph depicts the difference in salivary CERS1 levels in RAS patients and healthy individuals. There is a decrease in enzyme level of RAS patients (7.6 +/- 2.0 pg/ml) compared to healthy individuals (8.3 +/- 1.8 pg/ml). CERS1: Ceramide synthase 1; RAS: Recurrent aphthous stomatitis

We also observed a decrease in salivary CERS1 levels among RAS patients when compared with controls (Figure 2).

## Discussion

**Figure 3:**
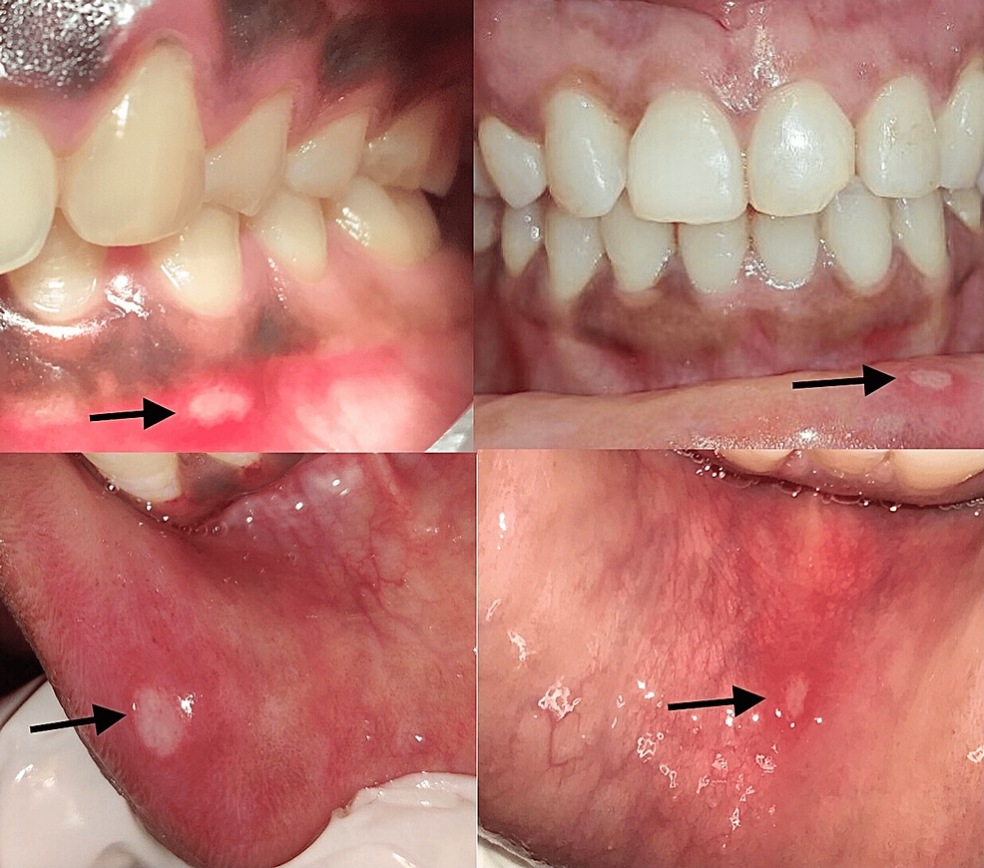
Clinical pictures Clinical pictures showing aphthous ulcers (arrows) involving various parts of the oral mucosa.

RAS presents with painful ulcers predominantly involving the non-keratinized oral mucosa, especially the lips and buccal mucosa (Figure 3).

RAS is predominantly seen in adolescents and young adults with a slight female predominance [1-3]. Our study participants were 22 to 32 years of age with a mean age of 27 years. We also observed 60% of RAS in females.

CERS1 is an enzyme that stimulates the synthesis of C18 CER and is found in the organelle membrane of the Golgi apparatus and endoplasmic reticulum [17]. In this study, we compared the levels of salivary CERS1 in healthy people and those with RAS. The investigation revealed that all of the included subjects showed CERS1 in their saliva. This demonstrates that CERS1 is necessary for preserving the oral microenvironment in healthy and diseased states. 

Radiation causes mucosal injury, that stimulates nuclear factor-kappa B (NF-Kb) and upregulation of injury-related apoptotic signals. These signals stimulate TNF-alpha, Interleukin (IL)-1 Beta, and IL-6. These mechanisms are mediated by CERS1, which leads to increased CER formation and hence an increase in the rate of apoptosis. This causes amplification of tissue injury leading to oral mucositis [17-20]. The mechanism of action for radiation-induced mucositis is similar to aphthous stomatitis in that they progress using similar pathways. In this study, we found that there was a decrease in the salivary CERS1 level in RAS patients compared to healthy individuals, but the result was statistically insignificant (p>0.05).

The pathogenic process of RAS is primarily cell-mediated, involving T-cells and TNF production [2]. The cytokines are one of the crucial elements that can trigger and choose the type of immune response in the human body. Th1-type cytokines, such as IL-2, IL-12, Interferon (IFN), and TNF, which also induce the cellular type response and stimulate immunoglobulin G (IgG) secretion, determine the propensity for autoimmunity. The anti-inflammatory properties of T-Helper Type (Th) 2-type cytokines, such as IL-4, IL-5, IL-10, and IL-13, stimulate the humoral immune response and immunoglobulin E (IgE) secretion [21,22]. Transforming growth factor (TGF), which is primarily secreted by the T-regulatory lymphocytes, also has a potent anti-inflammatory effect. It was discovered that RAS forms in response to an intensified immunologic response to specific areas of the oral mucosa. This reaction is attributed to a dysregulated cascade of cytokines and improper activate specific immune processes [23].

Th1 cytokine secretion was found to be significantly higher in RAS patients compared to controls. Both during the acute stage of the illness and during the period of remission, increased production of IL-2, IFN, and TNF was noted. When compared to healthy controls, the secretion of the anti-inflammatory cytokines TGF- and IL-10 was significantly reduced in patients with RAS [24]. This finding supports the hypothesis that in individuals who are predisposed to autoimmunity and aphthous stomatitis, an imbalance in the production of pro- and anti-inflammatory cytokines may play a role. In a study, the levels of IL-2 in healthy volunteers and patients with RAS were compared in peripheral blood and stimulated saliva. It has shown that RAS patients have significantly higher levels of this pro-inflammatory cytokine in their blood samples when compared to controls; however, stimulated saliva showed no significant differences [10,25].

Limitations

We observed that CERS1 was expressed in both controls and RAS cases. We also noticed a decrease in CERS1 levels in RAS cases. Hence, a larger multi-centric study including varying subtypes of RAS may yield a more comprehensive result.

## Conclusions

RAS is a painful ulceration that occurs in the oral cavity on a periodic basis. The etiology of this disease is still unknown. Several local, systemic, immunologic, genetic, allergic, nutritional, and microbial factors as well as immunosuppressive drugs have been proposed as causative agents.

Our study is the maiden attempt to evaluate the expression of salivary CERS1 in RAS. Further research is also needed to fully understand the intricate relationships of these CERs in controlling and interacting with the immune system.
